# Traditional health practitioners’ understanding of spirit possession in Gauteng province, South Africa

**DOI:** 10.4102/hsag.v29i0.1887

**Published:** 2024-03-21

**Authors:** Ellen M. Thobakgale, Roinah Ngunyulu, Mavis Mulaudzi

**Affiliations:** 1 Department of Nursing, Faculty of Health Sciences, Sefako Makgatho Health Sciences University, Ga-Rankuwa, South Africa; 2 Department of Nursing Science, Faculty of Health Sciences, University of Pretoria, Pretoria, South Africa; 3 Department of Nursing, Faculty of Health Care Sciences, University of Limpopo, Polokwane, South Africa; 4 Department of Nursing, Faculty of Health Care, University of Johannesburg, Johannesburg, South Africa

**Keywords:** spirit possession, understanding, phenomenology, hermeneutic, spiritual illness, traditional health practitioners, culture, religion

## Abstract

**Background:**

Traditional health practitioners (THPs) understand spirit possession as a cultural or religious spirit occupying a person, while the mental healthcare providers understand it as a mental illness. The different understanding is based on manifestations that mimic that of mental illness, such as seeing and hearing things that others cannot see or hear. Spirit possession holds different meanings in different cultures and religions that could be either beneficial or detrimental. Furthermore, spirit possession is understood as a channel of communication between the living and the dead or God or a supernatural phenomenon in which a spirit owns a person.

**Aim:**

This study explored and interpreted THPs’ understanding of spirit possession in Gauteng province, South Africa.

**Method:**

Hermeneutic phenomenology study explored and interpreted the THPs’ understanding of spirit possession in Gauteng province. In-depth individual interviews were conducted with 12 THPs who were selected through snowball sampling techniques. Data analysis followed Heidegger’s and Gadamer’s philosophies and Van Manen’s six steps of the analytic approach.

**Results:**

The findings revealed that THPs understood spirit possession as spiritual illness, ancestral calling and demonic spirit or witchcraft.

**Conclusion:**

Traditional health practitioners’ understanding of spirit possession could promote mental health and prevent mental illness by providing support to a spirit-possessed person and referral to mental healthcare services.

**Contribution:**

This study contributed that not all manifestations presented by persons with spirit possession are actual and clear-cut mental illness, but could be unwritten cultural and/ or religious illnesses that needs cultural and religious services also.

## Introduction

Spirit possession is a public occurrence found across 90% of the world’s population (Al-Adawi et al. 2019). In sub-Saharan Africa, the Circum-Mediterranean and North Africa, spirit possession has been reported to be higher at 81% (Boddy 1994). In Mozambique, Igreja et al. (2010) found a prevalence of 18.6% adults suffering from spirit possession. In Northern Uganda, 8% of the population suffered severe form of spirit possession (Neuner et al. 2012). Al-Adawi et al. (2019) described spirit possession as a concept that the spirit impersonates or possesses human being in which a person’s behaviour is thought to be controlled by a humanlike being that has entered a person’s body. According to Craffert (2015), spirit possession is a cultural technique with a variety of functions that is used to describe an illness or misfortune or an indication of a form of human dissociative phenomena. Rashed (2018) indicated that spirits are ghosts of departed ancestors or foreign visitors, divine beings, demons, and spirits of fire – in general, ethereal creatures of various origins. Furthermore, Rashed (2018) revealed that spirits inform people’s understanding of themselves and others as agency, responsibility, identity, normality and morality.

According to the traditional health practitioners (THPs) who are followers of traditional African religions, the illness can lead a spirit-possessed person as an individual to seek treatment and care from THPs than from mental healthcare services (Wayne 2014). In addition, family members prefer to take their spirit-possessed family members to THPs when the manifestations seemed to be that of spirit possession than mental illness. In this case, the individual’s physical, emotional, psychological, social and spiritual needs will be holistically attended to. Furthermore, it means that when the spirit-possessed person is holistically cared for, the family, the surrounding persons and/or the community are also taken care of in a cultural or religious manner.

Waterhouse (2017) supported the understanding of spirit possession as a condition that can often be cared and/or treated by cultural and/or spiritual methods beyond mental healthcare providers’ knowledge. However, there have been situations when the THPs realised that they were unable to assist their patients, they advised them to go to the local clinic and they made referrals for them for further management (Zuma et al. 2016). Such situations can be when the spirit-possessed person’s life or the lives of others are in danger because of the spirit-possessed person’s severe aggression, an eating disorder, dehydration or self-mutilation (American Psychiatric Association 2016).

Although the THPs understood spirit possession as a cultural and religious spirit, there was still a gap where spirit-possessed persons were treated and managed solely for cultural and/or religious spirit and ignoring the mental health aspect. Closing the gap can be made possible through training of the THPs and integrating them into the mental healthcare services for the benefits of mentally ill persons according to WHO-MHCAP 2013–2020 (Thobakgale 2021). Integrating the THPs in mental healthcare services can facilitate a two-way referral process that is from THPs to mental healthcare providers and vice versa (Zuma et al. 2016). Mental healthcare providers will educate THPs about manifestations of mental illness and decide which one they could treat and when to refer to the mental healthcare services (Sorsdahl & Stein 2013).

Researchers wondered how many spirit-possessed persons have been provided care by THPs as having either cultural or religious illness and not observed or identified as having clear-cut manifestations of mental illness. Not identifying clear-cut manifestations of mental illness can delay quality care, treatment and management of mental illness that could have been prevented at the primary level of care. Traditional health practitioners who can identify manifestations of mental illness during care of spirit-possessed persons are likely to immediately refer them to mental healthcare services. The study can assist THPs to acknowledge that mental illness does exist and at times it simultaneously exists with spirit possession. Mental illness needs mental healthcare services outside the THPs’ cultural and/or religious knowledge and understanding. Identifying the existence of mental illness in a spirit-possessed person is an attempt to provide holistic care and treatment of a spirit-possessed person’s psychological, social, emotional, spiritual and physical elements.

### Study aims

The aim of this study was to explore the THPs’ understanding of spirit possession in order to assist spirit-possessed persons to receive proper assessment and treatment that respect culture, religion or values and beliefs. This understanding can assist THPs to know when a spirit-possessed person needs mental healthcare services outside the cultural and religious services.

## Methods

The researchers used a hermeneutic phenomenological research approach to explore THPs’ understanding of spirit possession in order to bring to light and reveal upon the lived meaning of the spirit possession.

### Setting

The setting was in Gauteng province, South Africa, in the urban, peri-urban and farms. The study took place at the THPs’ healing places [*indumba*] or their homesteads in safe, neutral, quiet settings that were far from distractions and familiar to the participants. Some settings were old buildings far apart from each other in isolated bushes and on gravelled, sandy and dusty roads. Other settings were shacks while others were consulting rooms in their houses.

### Population

The population consisted of 12 THPs who had been providing care to spirit-possessed persons (see Table 1). Male and female THPs residing in Gauteng province, aged 29–75 years, who agreed and gave consent to participate in the study formed the study population. Traditional health practitioners who speak and understand Sepedi were included. Men and women who did not have an experience of taking care of the spirit-possessed persons, who did not agree to participate in the study, who could not speak and understand Sepedi and were residing outside the Gauteng province were excluded from the study.

**TABLE 1:** Demographic data of the traditional health practitioners.

Characteristics	Males (M)	Females (F)	Total number of THPs
Gender	4	8	12
Age	26-33 years	26-75 years	12
Race	Africans	Africans	12
Educational level	Grade 3 (1) Grade 4 (2) Diploma level (1)	Grade 4-11 (7) Matric (1)	12
Religion	Apostolic and/or African Initiative Religion (3) Charismatic (1)	Apostolic and/or African Initiative Religion (7) Charismatic (1)	12
Moths or years working with spirit-possessed person	Six month (1) From six months and above (3)	Six months and above (8)	12

*Source:* Thobakgale, E.M., 2021, ‘Spirit-possession as a mental illness: A phenomenological study in Gauteng province’, PhD thesis, Nursing Dept., University of Pretoria

THP, traditional health practitioner.

Twelve THPs who were known to have experiences in the care and treatment of spirit possession formed the study population. The sample size was reached by saturation when there was no new information received from THPs (Saunders et al. 2018).

### Sampling method and sample size

The researcher purposively sampled participants and used the snowball method to identify an information-rich population. The researcher contacted the first THP who recommended another THP. Thereafter, the researcher was referred to other THPs who had an opportunity to recommend another colleague until saturation was reached (Laverty 2008; Polit & Beck 2017). The sample size was guided by the saturation in hermeneutic (interpretive) phenomenology until the point of saturation with participant 7 (Laverty 2008; LoBiondo-Wood & Haber 2018). Furthermore, the researchers engaged in interviews with five more participants that ensured that no new information materialised (Saunders et al. 2018).

### Data collection

A phenomenological approach was followed to explore participants’ everyday life experience (Creswell 2014). The participants were reminded of the verbal and written agreement that was entered into prior to the actual interview. The researcher asked participants’ permission to use audio recordings to record the interviews (Pienaar 2016). In-depth interviews were used to collect data and probing questions were used to obtain more clarity and/or information following one-on-one interviews. The researcher also used observational skills as one of the data collection methods. Furthermore, the researcher used additional data sources to reflect on her thoughts about the interviews.

Interviews were conducted in both English and Sepedi, which are common languages spoken in Gauteng, South Africa. Interpretations were completed to accommodate participants who could only properly express themselves in Sepedi and/or felt uncomfortable with English (Thobakgale 2021). The data collection interview guide had one central research question (*What is your understanding of spirit possession?*) that facilitated the discussion. The participants (THPs) were interviewed at places suitable and familiar to them that were traditional consultation, healing places or homesteads. The researchers built rapport with participants which assisted in gaining trust and obtaining more information about their experiences before they became THPs.

The researcher observed some symbols and structures or type of structures, cloth(es), greetings, welcoming style, etc., that the participants used as explanations and motives to converse with (Russell-Bernard 2013; Van Manen 2014). Some participants were observed using interruptions of gestures, acts and sounds like *beb, yaah, eeei*, continuous yawning and clinching of hands among others.

### Data analysis

The researcher used the following concurrent procedural activities which involved six steps from Van Manen’s (2014) human science method of hermeneutic (interpretive) phenomenology (Lauterbach 2018; Thobakgale 2021; Van Manen 1990).

#### Step 1: Turning to the nature of the lived experience

The researcher familiarised herself with spirit possession as the phenomenon of interest, formulated the research question, clarified assumptions and pre-understanding.

#### Step 2: Investigating experience as we live it

The researcher captured the phenomenon through various methods of investigation:

- used interviews and personal experience as a starting point
- traced etymological sources
- searched idiomatic phrases
- obtained experiential descriptions from THPs
- observed emotions
- consulted phenomenological literature, experiential descriptions in literature, life story as a resource for experiential material, diary and journal.

#### Step 3: Phenomenological writing or describing the phenomenon in the art of writing and rewriting

The researcher made the feelings, thoughts and attitudes of the participants visible through the process of writing. The researcher paid more attention to THPs’ spoken language and thoughts of their understanding of spirit possession. The researcher created a table of all the thematic statements and themes, then wrote notes and paragraphs on each to capture the themes. The researcher, study promoter and co-promoter constantly deliberated repeated themes at consistent intervals until final themes were derived (see Table 2).

**TABLE 2:** Summary of main themes and sub-themes from traditional health practitioners.

Main themes		Sub-themes
1.	Understanding spirit possession	1.1 Spirit possession as spiritual illness [*Bolwetši bja semoya*] 1.2 Spirit possession as a spiritual calling [*Pitšo ya semoya*] 1.3 Spirit possession as witchcraft [*Boloi*]

*Source:* Adapted from Thobakgale, E.M., 2021, ‘Spirit-possession as a mental illness: A phenomenological study in Gauteng province’, PhD thesis, Nursing Dept., University of Pretoria

#### Step 4: Phenomenological reflection or reflecting on the essential themes, which characterise the phenomenon

The overall meaning of a participant’s experience was sought when reflecting on the themes. The researchers conducted thematic analysis on situations to seek meaning and reflective notes assisted this.

#### Step 5: Maintaining a strong and oriented position to the phenomenon

To maintain a strong and oriented position on spirit possession, the researchers used a journal of reflection to refocus on the research question and kept frequent contact with colleagues and supervisors.

#### Step 6: Balancing the research context by considering the parts and the whole

The researcher continually measured the inclusive design of the study or text in contradiction of the significance that the parts had to play in the total textual structure. The researcher applied back and forth movement using the six steps that flowed one into the other in a spiral way. Then, data were combined in a sequential back and forth movement with the aim of looking at the whole picture to ensure that all the different parts contributed to the production of a complete picture. The researcher used the process of back and forth during reading and re-reading to analyse and interpret data in a circular form (Lauterbach 2018). The researcher analysed the interview transcripts immediately after the first data collection.

### Ethical considerations

Ethical clearance to conduct this study was obtained from the Faculty of Health Sciences Research Ethics Committee Department of Nursing Science In-House Committee, School of Health Care Sciences Post-Graduate Committee, Department of Health in the Gauteng province and the National Health Research Database (No. 201/2018).

## Review findings

The study findings revealed demographic data (see Table 1) of the THPs and the understanding of spirit possession as either spiritual illness, spiritual calling or witchcraft (see Table 2).

The majority of participants were female THPs in Gauteng province (67%). In total, there were 12 THPs aged from 29 years to 75 years. Eight women and four men who had experience in the care and treatment of spirit-possessed persons. The age for the male participants ranged between 29 years and 33, and their educational status was from Grade 3 to diploma level. Four THPs belonged to the Apostolic Religion-African Initiative Churches; one belonged to the charismatic faith and seven of them belonged to a traditional religion. The eight female THPs were Africans whose age ranged from 29 years to 75 years of age. Their educational status ranged from grades 7 to grade 12.

### Sub-theme 1.1: Spirit possession as a spiritual illness [*Bolwetši bja semoya*]

The understanding was that, when a person rejected their calling from their ancestors, the ancestors visited a person who was likely to present unfamiliar behaviour like talking, laughing, crying, outbursts, anger and/or dancing alone. Some spirit-possessed persons experienced dreams or visions that were frightening as evidenced by THPs in the following quotes:

‘[*A*]ncestral spirit does make a person’s mind not to function, however, the person will talk with ancestor in the language that people around them does not understand.’ (THP1, F, 55 years old)

One participant mentioned that spirit possession manifested in terrifying dreams and visions. In a way, the manifestations and the dreams guided the THP to determine if it was a spiritual illness [*bolwetši bja semoya*] or something else. The participant said:

‘I can say spirit-possession refers to a person with dreams of either water, have visions where [*s*]he has been chased in the river, visions on snakes, dreams or visions of grand or great-grand parents and sometimes see them in the water.’ (THP2, F, 42 years old)

Another participant echoed that spiritual illness could be experienced when the spirit-possessed person experiences or senses the suffering of the other person whom the ancestors wanted to reveal such as:

‘[*S*]ome other signs among others are that what I experienced is what the person I am staying with or closer to me suffers.’ (THP7, M, 35 years old)

### Sub-theme 1.2: As spiritual calling [*Pitšo ya semoya*]

Spiritual calling from the ancestors that led a person to be a diviner or prophet through a process called *ukuthwasa*. ‘Spiritual calling’ was in most cases used interchangeably with the term ‘gift’. The gift was not an individual’s choice but as a chosen family member to carry on with the work that ancestors were doing. Sometimes if the spirit-possessed person’s parents or immediate family members refused to take up the gift, then the calling befell the other descendants. The quotes below evidenced that spirit possession was a spiritual calling or gift that presented itself in different ways, leading a person to follow the calling to become a THP:

‘This calling can be an ancestral or religious calling or *ukuthwasa* where one has to be trained.’ (THP1,F, 55 years old)

A participant indicated that:

‘Bongaka [*traditional healing*] is a gift. People may look down upon healing and yet is what you inherit. If they chose you to be a traditional health practitioner, you will be and this is the spirit of the dead that may be your ancestors wants [*sic*] you to serve people.’ (THP2, F, 42 years old)

Another participant said the calling differed according to the possessing spirit:

‘[*A*]mongst the spiritual gifts there is a prophetic gift and healing gift and all these gifts are from the ancestors.’ (THP4, M, 46 years old)

To show that the spirit possession was a gift, the spirit-possessed person would have some revelation in their dreams of a light sleep, vision or have some senses on the body as confirmed by the quotes below: ‘dreaming [*of*] herself under the water, digging muti, crushing muti or dreaming [*of*] herself in some ancestral clothes.

One participant indicated that some spirit-possessed persons were gifted with different powers:

‘[*I*]t means a person is gifted to see, feel, hear things that are not always experienced by some church members.’ (THP7, M, 35 years old)

‘in the vision the person may be informed that this is the time to connect with your [*ancestors*]. The person may see prophetic clothing such as cape-like coat, white, red or any colours of cloth that needs to be put on the head.’ (THP9, F, 67 years old)

### Sub-theme 1.3: Spirit possession as witchcraft [*Boloi*]

Traditional health practitioners understood witchcraft as the practice of evil acts wherein witches inflicted a person with an evil spirit that ruined that person’s life. The infliction of the evil spirit could come from family members and commonly caused a person to have the spirit called *lefofonyane* or *mafonfonyane. Mafonfonyane* was an evil spirit that presented itself like mental illness. Also, witchcraft was understood as a practice where witches bewitched books so that when people read these titles their minds became affected. Some were initiated during the night so that they could start practising witchcraft; other witches spoke evil into the soil so that bad things happened to the targeted person:

‘[*S*]ometimes the person can be bewitched. The witches can be within the family or someone unrelated. The spirit-possessed person may experience lefofonyane where a persons’ eyes looks [*sic*] up and the white part becomes … visible.’ (THP5, F, 52 years old)

‘[*S*]pirit-possession mean those people are possessed by useless spirit which drives them to do wrong.’ (THP10, F, 69 years old)

‘[*C*]apture by other forces like the inflictions from other people and the person is not free or witches bewitch a book or prick down a book with a needle to possess or capture the mind of a person. Some wake up and do miracles, some witches initiate persons or connect them to the moons or stars. The same happens if people take the soil and talk bad into that soil, the predictions will happen.’ (TPH11, F, 67 years old)

The process of witchcraft in terms of taking soil and speaking bad about a person was common when one talked at a graveside and wished bad luck upon others.

## Discussion of findings

Traditional health practitioners understood and described spirit possession as spiritual illness [*bolwetši bja semoya*], spiritual calling [*pitšo ya semoya*] and witchcraft [*boloi*]. According to Khan and Sahni (2013), spirit possession was a supernatural phenomenon or illness in which a spirit, demon, extra-terrestrial being or disincarnate subject, including God, possessed a person. There were noticeable changes in a person, such as ill health, changes in behaviour or appearance. According to Mothibe and Sibanda (2019), a person was spirit-possessed if he or she was occupied by the ancestral spirit that used them as a channel for communication between the living and the dead.

### Spirit possession as a spiritual illness [*Bolwetši bja semoya*]

Traditional health practitioners understood spirit possession as a spiritual illness or ancestral illness that was attributable to supernatural forces such as ancestral spirits, harmless household spirits, wild forest spirits, malevolent spirits and shaman spirits. The severity of the illness depended on the number of spirits that possessed a person.

The person with spiritual illness behaved in a mentally deranged way and sometimes presented psychotic features fuelled by the spirits associated with witches and sorcerers. Some persons with spiritual illness experienced traumatic dreams while others experienced tremors, voices, visions, and so on. The person became stressed, angry and/or isolated and ultimately ended up in a mental healthcare institution because of unresolved spiritual illness. The spirit-possessed person likely felt worthless, lost hope and developed suicidal ideas that were also regarded as signs of mental illness.

Zuma et al. (2016) and Ndlovu (2016) described spirit possession as a spiritual illness that revealed itself in a person through a combination of physical, emotional or psychological illnesses. Mokgobi (2014), Lebaka (2018) and Sodi (1998) reported ritualistic dancing and singing/*malopo* dancing or therapeutic dancing as another type of spiritual illness. Xaso (2015) and Booi (2004) echoed that the spirit-possessed person with spiritual illness was in most cases labelled as suffering from mental illness or physical ailments that could not be detected by western methods. Spiritual illness was a state of the soul in which interpretation, and reflexivity came together to produce effects of frustration in a person’s life (Neuhouser 2014). Moffat (2011) described spiritual illness as an indigenous mental illness that was deeply connected to spirituality and cultural beliefs. Kpobi and Swartz (2018) understood spiritual illness as supernatural factors that could cause an intellectual ill health.

### Spirit possession as a spiritual calling [*Pitšo ya semoya*]

Traditional health practitioners understood spirit possession as a calling from the ancestors or angels. The calling could be from grandparents who have passed on and depending on the patient’s cultural or religious background, they were called ancestors or angels. Here, THPs used ‘a calling’ interchangeably with ‘a gift’ from the ancestors, God or angels. A spirit-possessed person with a spiritual calling portrayed either a gift of prophecy, healing or divination, while others were gifted with all three. Further, THPs described how a person with spiritual calling entered dialogue or communication with the possessing ancestors, through the senses (e.g., touch, sight, etc.). A calling could be either for *ukuthwasa*, prophecy or both. Palmer’s (2014) revelation supported that spirit possession was a calling by indicating that THPs (faith healers) were possessed by the Holy Spirit and (trance mediums) were possessed by their spiritual ‘guides’.

### Spirit possession as witchcraft [*Boloi*]

Traditional health practitioners understood spirit possession as the practice of witchcraft (*boloi*). The terms ‘demon’ and ‘evil possession’ were used interchangeably with witchcraft [*boloi*]. According to THPs, witches inflict unwanted objects using magical spells to disrupt a person’s life. The spirit-possessed person likely displayed manifestations that were like those observed in mental illness.

Three types of witchcraft were described and understood: firstly, manipulating objects in nature as well as through incantations, charms and spells to harm others; secondly, witchcraft meant engaging in sexual relations with the devil in exchange for supernatural powers; and thirdly, witches shared common goals especially in assisting each other in harming their enemies (Ally 2015). Semenya and Letsosa (2012) revealed that witchcraft was an act of evil and potentially hurtful to people. Witchcraft or demonic power operated from the person whom the witches had possessed in the sense that a person’s normal way of living was disrupted wherein the demonic powers spoke and acted through the human being as their complete slave and instrument (Gehman 1989; Juro 2016).

## Implications and recommendations

This study has implications for policymakers on mental health in general and THPs who take care of spirit-possessed persons. Traditional health practitioners need to take note of the results and treat spirit-possessed person for what they understand as cultural and/or religious issues and only then refer for conditions that they do not understand. Evidence from the study reports THPs’ views that spirit possession is not a mental illness but a spiritual illness, an ancestral calling and/or demonic spirit or witchcraft. Further research is recommended to explore the manifestations in spirit-possessed person that could reveal mental illness. It is therefore recommended that THPs should be engaged and assist healthcare providers with other illnesses outside mental health parameters. Engagement could be helpful as it may reveal issues that could guide policymakers in the mental health services. Involving THPs could also advance pathways to mental healthcare services from primary healthcare settings in order to assist spirit-possessed persons to receive a comprehensive care combining physical, spiritual, emotional and psychosocial elements.

Culture and religious practices must be addressed in the curriculum of mental healthcare providers and in-service training by experts should be provided in order to embrace the values, morals and world views of the spiritual illnesses that are not listed in the current mental health diagnostic instrument such as Diagnostic and Statistical Manual of Mental Disorders, fifth edition (DSM 5).

## Conclusion

From the findings of this study, it is clear that THPs seemingly understood spirit possession as spiritual illness, spiritual calling and witchcraft. It is implied that the THPs need a better understanding of mental healthcare services approaches to the care and treatment of mental illness, while mental healthcare providers also need to be orientated about a culture and religion-centred approach to mental healthcare.

## References

[CIT0001] Al-Adawi, S., Al-Kalbani, Y., Panchatcharam, S.M., Al-Zadjali, M.A., Al-Adawi, S.S., Essa, M.M. et al., 2019, ‘Differential executive functioning in the topology of Spirit possession or dissociative disorders: An explorative cultural study’, *BMC Psychiatry* 19, 379. 10.1186/s12888-019-2358-2 31791283 PMC6889563

[CIT0002] Ally, Y., 2015, ‘Burn the witch: The impact of the fear of witchcraft on social cohesion in South Africa’, *Psychology in Society* 49, 25–45. 10.17159/2309-8708/2015/n49a3

[CIT0003] American Psychiatric Association, 2016, ‘APA updates guidelines on psychiatric evaluation in adults’, *Practice Guidelines* 94(1), 62–64. 10.1176/appi.books.9780890426760

[CIT0004] Booi, N.B., 2004, ‘Three perspectives on ukuthwasa: The view from traditional beliefs, western psychiatry and transpersonal psychology’, Master’s thesis, Clinical Psychology Depart, Rhodes University.

[CIT0005] Boddy, J., 1994, ‘Spirit possession revisited: Beyond instrumentality’, *Annual Review of Anthropology* 23, 407–434. 10.1146/annurev.an.23.100194.002203

[CIT0006] Craffert, P.F., 2015, ‘What does it mean to be possessed by a spirit or demon? Some phenomenological insights from neuro-anthropological research’, *HTS Teologiese Studies/Theological Studies* 71(1), 2891. 10.4102/hts.v71i1.2891

[CIT0007] Creswell, J.W., 2014, *Research design qualitative, quantitative and mixed methods approaches*, Sage, London. 10.7748/nr.12.1.82.s2 28718745

[CIT0008] Igreja, V., Dias-Lambranca, B., Hershey, D.A., Racin, L., Richters, A. & Reis, R., 2010, ‘The epidemiology of spirit possession in the aftermath of mass political violence in Mozambique’, *Social Science & Medicine* 71, 592–599. 10.1016/j.socscimed.2010.04.024 20542612

[CIT0009] Gehman, R.J., 1989, *African traditional religion in biblical perspectives*, Easy Africa Publishers, Nairobi.

[CIT0010] Juro, C., 2016, ‘A church historical judicial assessment of the Reformed Church in Zimbabwe engagement with demon possession and exorcism’, Master’s thesis, Dept of Theology, Stellenbosch University.

[CIT0011] Khan, I.D. & Sahni, A.K., 2013, ‘Possession at high altitude’, *Kathmandu, University Medical Journal* 11(3), 253–55. 10.3126/kumj.v11i3.12516 24442177

[CIT0012] Kpobi, L. & Swartz, L., 2018, ‘Implications of healing power and positioning for collaboration between formal mental health services and traditional/alternative medicine: The case of Ghana’, *Global Health Action* 11, 1. 10.1080/16549716.2018.1445333 29529937 PMC5912442

[CIT0013] Lauterbach, A.A., 2018, ‘Hermeneutic phenomenological interviewing: Going beyond semi-structured formats to help participants revisit experience’, *The Qualitative Report* 23(11), 2883. 10.46743/2160-3715/2018.3464

[CIT0014] Laverty, S.M., 2008, ‘Hermeneutic phenomenology and phenomenology: A comparison of historical and methodological considerations’, *International Journal of Qualitative Methods* 2(3), 21–35. 10.1177/160940690300200303

[CIT0015] Lebaka, M.E.K., 2018, ‘The art of establishing and maintaining contact with ancestors: A study of Bapedi tradition’, *HTS Teologiese Studies/Theological Studies* 74(1), 4871. 10.4102/hts.v74i1.4871

[CIT0016] LoBiondo-Wood, G. & Haber, J., 2018, *Nursing research: Methods and critical appraisal for evidence-based practice*, Mosby, New York, NY.

[CIT0017] Moffat, L.L., 2011, ‘Mental illness or spiritual illness: What should we call it?’, *MJA* 194(10), 541–542. 10.5694/j.1326-5377.2011.tb03097.x 21644908

[CIT0018] Mokgobi, M.G., 2014, ‘Understanding traditional African healing’, *African Journal for Physical Health Education, Recreation and Dance (AJPHERD)* 20(2), 24–34. 26594664 PMC4651463

[CIT0019] Mothibe, M.E. & Sibanda, M., 2019, *African traditional medicine: South African perspective*, Iintechopen, Pretoria.

[CIT0020] Ndlovu, S.S.P., 2016, ‘Traditional healing in KwaZulu-Natal Province: A study of University students’ assessment, perceptions and attitudes’, Master’s thesis, School of Applied Human Sciences, University of KwaZulu-Natal.

[CIT0021] Neuhouser, F., 2014, ‘Nietzsche on spiritual illness and its promise’, *Journal of Nietzsche Studies* 45(3), 293–314. 10.5325/jnietstud.45.3.0293

[CIT0022] Neuner, F., Pfeiffer, A., Schauer-Kaiser, E., Odenwald, M., Elbert, T., Ertl, V., 2012, ‘Haunted by ghosts: Prevalence, predictors and outcomes of spirit possession experiences among former child soldiers and war-affected civilians in Northern Uganda’, *Social Science & Medicine* 75, 548–554. 10.1016/j.socscimed.2012.03.028 22580073

[CIT0023] Palmer, A., 2014, *Principles of services marketing*, McGraw-Hill Education, Hounslow.

[CIT0024] Pienaar, M., 2016, ‘Narratives of partners of people diagnosed with bipolar disorder’, Master’s thesis, Psychology Dept., University of the Witwatersrand.

[CIT0025] Polit, D.F. & Beck, C.T., 2017, *Nursing research: Generating and assessing evidence for nursing practice*, Lippincott Williams & Wilkins, Philadelphia, PA.

[CIT0026] Rashed, M.A., 2018, ‘More things in heaven and earth: Spirit possession, mental disorder, and intentionality’, *Journal of Medical Humanities* 41(3), 363–378. 10.1007/s10912-018-9519-z PMC7343734 30027384

[CIT0027] Russell-Bernard, H., 2013, *Social research methods qualitative and quantitative approaches*, University of Florida, Gainesville, FL.

[CIT0028] Saunders, B., Sim, J., Kingstone, T., Baker, S., Waterfield, J., Bartlam, B. et al., 2018, ‘Saturation in qualitative research: Exploring its conceptualization and operationalization’, *Quality & Quantity* 52(4), 1893–1907. 10.1007/s11135-017-0574-8 29937585 PMC5993836

[CIT0029] Semenya, D.K. & Letsosa, R., 2012, ‘Biblical principles as an answer to the African people’s questioning of witchcraft’, *Verbum et Ecclesia* 33(1), 674. 10.4102/ve.v33i1.674

[CIT0030] Sodi, T., 1998, ‘A phenomenological study of healing in a north Sotho community’, PhD thesis, Dept. of Psychology, University of Cape Town.

[CIT0031] Sorsdahl, K. & Stein, D.J., 2013, ‘Predicting referral practices of traditional healers of their patients with a mental illness: An application of the theory of planned behaviour’, *African Journal of Psychiatry* 16(1), 35–40. 10.4314/ajpsy.v16i1.6 23417634

[CIT0032] Thobakgale, E.M., 2021, ‘Spirit-possession as a mental illness: A phenomenological study in Gauteng province’, PhD thesis, Nursing Dept., University of Pretoria.

[CIT0033] Van Manen, M., 1990, *Researching lived experience: Human science for an action sensitive pedagogy*, State University of New York Press, Albany, NY.

[CIT0034] Van Manen, M., 2014, *Phenomenology of practice: Meaning-giving methods in phenomenological research and writing*, Left Coast Press, Walnut Creek, CA.

[CIT0035] Waterhouse, S., 2017, *Strength for His people*, West cliff Bible Church, Amarillo, TX.

[CIT0036] Xaso, Z.C., 2015, ‘The meaning of ukuthwasa: Urban youth perspectives on social change and the persistence of tradition in the Eastern Cape’, Master’s thesis, Dept. of African studies, University of Fort Hare.

[CIT0037] Zuma, T., Wight, D., Rochat, T. & Moshabela, M., 2016, ‘The role of traditional health practitioners in Rural KwaZulu-Natal, South Africa: Generic or mode specific?’, *BMC Complementary and Alternative Medicine* 16, 304. 10.1186/s12906-016-1293-8 27549895 PMC4994274

